# Prognostic value of Lynch syndrome, *BRAF^V600E^*, and *RAS* mutational status in dMMR/MSI‐H metastatic colorectal cancer in a pooled analysis of Dutch and French cohorts

**DOI:** 10.1002/cam4.6223

**Published:** 2023-06-16

**Authors:** Koen Zwart, Frederieke H. van der Baan, Romain Cohen, Thomas Aparicio, Christelle de la Fouchardiére, Thierry Lecomte, Cornelis J. A. Punt, David Sefrioui, Rik J. Verheijden, Geraldine R. Vink, G. Emerens Wensink, Aziz Zaanan, Miriam Koopman, David Tougeron, Jeanine M. L. Roodhart

**Affiliations:** ^1^ Department of Medical Oncology, University Medical Center Utrecht Utrecht University Utrecht The Netherlands; ^2^ Department of Epidemiology, Julius Center for Health Sciences and Primary Care University Medical Center Utrecht Utrecht The Netherlands; ^3^ Department of Medical Oncology, Équipe Instabilité des Microsatellites et Cancer, Équipe Labellisée par la Ligue Nationale Contre le Cancer et SIRIC CURAMUS Centre de recherche Saint Antoine, Hôpital Saint‐Antoine, AP‐HP, and INSERM UMRS 938, Sorbonne Université Paris France; ^4^ Gastroenterology Department, Saint Louis Hospital, AP‐HP University of Paris Paris France; ^5^ Gastroenterology Department Avicenne Hospital Bobigny France; ^6^ Medical Oncology Department Léon Bérard Center Lyon France; ^7^ Department of Hepato‐Gastroenterology and Digestive Oncology, Tours University Hospital and INSERM UMR 1069 N2C University of Tours Tours France; ^8^ Digestive Oncology Unit, Department of Hepatogastroenterology, Rouen University Hospital, IRON Group and INSERM U1245 University of Normandy Rouen France; ^9^ Department of Research and Development Netherlands Comprehensive Cancer Organisation (IKNL) Utrecht The Netherlands; ^10^ Department of Gastroenterology and Digestive Oncology, Georges Pompidou European Hospital Assistance publique–Hôpitaux de Paris, SIRIC CARPEM, University Paris Cité Paris France; ^11^ Hepato‐Gastroenterology Department Poitiers University Hospital, University of Poitiers Poitiers France

**Keywords:** deficient mismatch repair, Lynch syndrome, metastatic colorectal cancer, microsatellite instability, molecular biology

## Abstract

**Background:**

Current knowledge on prognostic biomarkers (especially *BRAF* ^*V600E*^/*RAS* mutations) in metastatic colorectal cancer (mCRC) is mainly based on mCRC patients with proficient mismatch repair (pMMR) tumors. It is uncertain whether these biomarkers have the same prognostic value in mCRC patients with deficient mismatch repair (dMMR) tumors.

**Methods:**

This observational cohort study combined a population‐based Dutch cohort (2014–2019) and a large French multicenter cohort (2007–2017). All mCRC patients with a histologically proven dMMR tumor were included.

**Results:**

In our real‐world data cohort of 707 dMMR mCRC patients, 438 patients were treated with first‐line palliative systemic chemotherapy. Mean age of first‐line treated patients was 61.9 years, 49% were male, and 40% had Lynch syndrome. *BRAF* ^*V600E*^ mutation was present in 47% of tumors and 30% harbored a *RAS* mutation. Multivariable regression analysis on OS showed significant hazard rates (HR) for known prognostic factors as age and performance status, however showed no significance for Lynch syndrome (HR: 1.07, 95% CI: 0.66–1.72), *BRAF* ^*V600E*^ mutational status (HR: 1.02, 95% CI: 0.67–1.54), and *RAS* mutational status (HR: 1.01, 95% CI: 0.64–1.59), with similar results for PFS.

**Conclusion:**

*BRAF* ^*V600E*^ and *RAS* mutational status are not associated with prognosis in dMMR mCRC patients, in contrast to pMMR mCRC patients. Lynch syndrome is also not an independent prognostic factor for survival. These findings underline that prognostic factors of patients with dMMR mCRC are different of those with pMMR, which could be taken into consideration when prognosis is used for clinical decision‐making in dMMR mCRC patients and underline the complex heterogeneity of mCRC.

## Graphical abstract

An original research article to provide insight in the prognostic value of Lynch syndrome, BRAF^V600E^ and RAS mutation status on survival outcomes with pooled individual patient data from the large Dutch and French deficient mismatch repair (dMMR) / microsatellite instability‐high (MSI‐H) metastatic colorectal cancer (mCRC) cohort. Lynch syndrome, BRAF^V600E^ and RAS mutational status are not independently associated with overall survival in dMMR/MSI‐H mCRC patients treated with palliative first‐line chemotherapy, in contrast to proficient MMR mCRC patients. This could be taken into consideration when prognosis is used for clinical decision‐making in dMMR mCRC patients and underline the complex heterogeneity of mCRC.

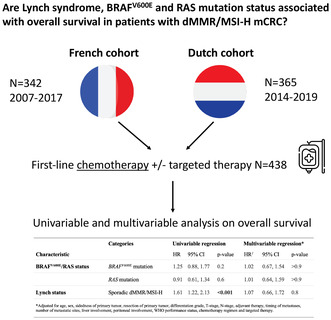

## 1 INTRODUCTION

Colorectal cancer (CRC) is a heterogeneous disease characterized by different genomic landscapes and carcinogenic pathways.^1^, ^2^ One of the carcinogenic pathways is microsatellite instability (MSI) due to deficient DNA mismatch repair (dMMR).^3^ This feature is present in approximately 15%–20% of patients with early‐stage CRC and 3%–5% of patients with metastatic CRC (mCRC).^4^, ^5^, ^6^ The origin of dMMR can be due to inherited germline defects in patients with Lynch syndrome (constitutional mutation of one MMR gene), also known as hereditary non‐polyposis colorectal cancer (HNPCC), or sporadic, mostly by aberrant hypermethylation and epigenetic silencing of *MLH1* gene.^3^

The heterogeneity of CRC is expressed by many molecularly‐defined subgroups with differences in response to treatment and prognosis.^7^, ^8^ This knowledge is mainly based on CRC patients with proficient mismatch repair/microsatellite stable (pMMR/MSS) tumors, for which *RAS* and *BRAF* ^*V600E*^ mutations are well‐established predictive and prognostic biomarkers.^9^, ^10^ Both *RAS* and *BRAF* ^*V600E*^ mutations, but particularly *BRAF* ^*V600E*^ mutations, are associated with inferior progression‐free survival (PFS) and overall survival (OS) in mCRC patients.^10^ Tumors with a *RAS* mutation are resistant to treatment with anti‐epidermal growth factor receptor (anti‐EGFR) therapy. *BRAF* ^*V600E*^ mutated tumors can be effectively treated with a combination of encorafenib (*BRAF* inhibitor) plus cetuximab (anti‐EGFR).^11^, ^12^ However, the prognostic value of these biomarkers has not been investigated in a large cohort of patients with dMMR/MSI‐H mCRC, so the prognostic value of these biomarkers in this population remains uncertain.^4^, ^13^, ^14^, ^15^, ^16^, ^17^, ^18^

Immune checkpoint inhibitors (ICIs) have shown a marked improvement in PFS and OS in patients with dMMR/MSI‐H mCRC.^19^, ^20^ However, standard systemic therapy remains an important treatment option in these patients.^21^ In a large randomized controlled trial, primary resistance to ICI occurred in 30% of patients and more than 50% of patients required second‐line treatment with systemic chemotherapy with or without targeted therapy.^19^ However, chemotherapy and targeted therapy may have different efficacy in patients with dMMR/MSI‐H CRCs compared to pMMR/MSS CRCs, as has been shown with adjuvant chemotherapy in the stage II and III setting with resistance to fluoropyrimidine monotherapy.^22^, ^23^, ^24^ This underlines the need to examine the use of different treatment regimens in patients with dMMR/MSI‐H tumors within the metastatic setting.^25^, ^26^

Data of mCRC patients with dMMR/MSI‐H tumors are scarce because of the low incidence in the metastatic setting and previous studies show conflicting results regarding prognosis of the *BRAF* ^*V600E*^ mutation and uncertain results of Lynch syndrome and RAS mutations due to low number of included patiens with dMMR/MSI‐H mCRC.^4^, ^13^, ^14^, ^15^, ^16^, ^17^, ^18^ International collaborations with real‐world data are needed to enable a large enough cohort to evaluate prognostic factors and predictive factors of treatment response.^4^, ^7^ Identifying subgroups within the dMMR/MSI‐H mCRC population is of importance for clinical decision‐making and knowledge of effective treatment regimens that could improve survival. The aim of this cohort of dMMR/MSI‐H mCRC patients is to provide insight in the prognostic value of Lynch syndrome, *BRAF* ^*V600E*^ and *RAS* mutation status and the effect of treatment regimens on survival outcomes with pooled individual patient data from the largest Dutch and French dMMR/MSI‐H mCRC cohorts up until now.

## 2 METHODS

### 2.1 Study population and data collection

This observational cohort study combined a nationwide population‐based Dutch cohort and a French multicenter observational cohort of adult dMMR/MSI‐H mCRC patients. For the Dutch cohort individual data were collected in the period of 2014–2019 by well‐trained data managers of the Netherlands Comprehensive Cancer Organization (IKNL) and registered in the Netherlands Cancer Registry (NCR). Data collection included all Dutch centers by linkage with the Dutch Nationwide Pathology Databank (PALGA), thereby capturing every patient with histologically proven CRC.^27^ This linkage was also used to obtain all original pathology excerpts, including *BRAF* ^*V600E*^ (c.1799 T > A, p.V600E) and *RAS* status, if determined during daily clinical practice. Status of *BRAF* ^*V600E*^ and RAS was established by next generation sequencing, according to national guidelines, in almost all cases. The NCR data were pseudonomized and consent was obtained by an opt‐out approach. Data of the French cohort was collected in the period of 2007–2017 in 18 French centers by local physicians and/or clinical research associates.^15^ The French data collection was approved by the ethical committee *Comité de Protection des Personnes Ouest III* and, due to the retrospective nature of the study and since most patients were deceased, informed consent was waived.

In both cohorts, all consecutive patients with histologically proven dMMR and/or MSI‐H mCRC were included. Patients with a concurrent malignancy interfering with the prognosis and patients with short follow‐up (≤15 days) were excluded. In addition, inconclusive cases with discordance between MSI and MMR immunohistochemistry status (MSI/pMMR or MSS/dMMR) were not included in the study.

### 2.2 Deficient mismatch repair and microsatellite instability

MMR and/or MSI status was only known if tested in daily clinical practice and was obtained by analysis of the four MMR proteins expression by immunohistochemistry and/or DNA MSI testing in accredited laboratories according to international guidelines.^28^ MMR expression was defined as deficient when there was a nuclear loss in protein expression of either MLH1, PMS2, MSH2 or MSH6 proteins. In the French cohort MSI was assessed with the mononucleotide repeat pentaplex panel (BAT‐25, BAT‐26, NR‐21, NR‐22, and NR‐24) and was determined as MSI‐H when at least three markers showed microsatellite instability. In the Dutch cohort MSI was assessed with the mononucleotide repeat pentaplex panel (BAT‐25, BAT‐26, NR‐21, MONO‐27 and NR‐24) and determined as MSI‐H when at least two markers showed microsatellite instability.

Whether patients were identified with Lynch syndrome or sporadic dMMR/MSI‐H was based on a tailored approach by MMR protein expression, family history, *BRAF* ^*V600E*^ status and *MLH1* promotor hypermethylation status (details available in supplements) in both the Dutch and the French cohort, as previously described.^15^, ^29^

### 2.3 Outcome

In the Dutch cohort, the NCR was linked to the National Municipal Personal Records Database in January 2021 to obtain the most recent information on vital status. In the French cohort, the vital status was updated until September 2019. OS was defined as treatment initiation of palliative first‐line (OS1), second‐line (OS2) or third‐line (OS3) treatment until death. PFS was defined as survival from treatment initiation of first‐line (PFS1), second‐line (PFS2) or third‐line (PFS3) treatment until progression or death, whichever occurred first.

A new line of therapy was defined when a new systemic therapy was initiated, including change of therapy due to toxicity or progression. It was not considered an event for PFS if a new line of therapy was initiated without documented progression or death. Adjuvant therapy was only considered a line of therapy when progression occurred during the adjuvant chemotherapy or within 6 months after start of treatment. Patients treated with ICIs and non‐standard chemotherapy (e.g., experimental systemic therapy) were only included for analyses in treatment lines preceding these therapies. Sensitivity analyses were performed with censoring of these patients at the start of ICI and/or local treatment.

### 2.4 Statistical analysis

Median follow‐up time was analyzed with reversed Kaplan–Meier analyses. The primary endpoint was survival on first‐line treatment, for which PFS1 and OS1 were analyzed. Kaplan–Meier curves were obtained for univariable analysis, and Cox regression analysis was used for multivariable analysis. Patients were censored at date of last follow‐up for patients alive and without disease progression.

Cox proportional hazard regression models with 95% confidence intervals (CIs) included preselected factors, based on literature and expert opinion: age at diagnosis mCRC, sex, sidedness of primary tumor (right‐sided, defined as cecum to transverse colon, left‐sided, defined as splenic flexure to sigmoid, and rectum), primary tumor resection, grade, T‐stage, N‐stage, adjuvant therapy, metachronous or synchronous mCRC (synchronous was defined as the diagnosis of a distant metastasis within 6 months of the diagnosis of primary CRC^30^), number of metastatic sites, liver involvement, peritoneal involvement, *BRAF* ^*V600E*^ and *RAS* mutational status, Lynch syndrome status, World Health Organization performance score (WHO PS), chemotherapy regimen, targeted therapy and with stratification for country.^30^, ^31^ The proportional hazard assumption was visually examined with Schoenfeld residuals. Multiple imputation by substantive model compatible fully conditional specification (SMC‐FCS) was used for missing data.^32^ The variables used for imputation were the same as for the Cox regression model. Regression analyses were performed on each imputed dataset and HR were combined with Rubin's rules.

To study chemosensitivity, PFS analyses were restricted to first‐line patients. Subgroups for molecular status and Lynch syndrome status were analyzed when at least 20 patients were present in each of the arms. Additionally, PFS and OS analyses were performed from start second‐line and third‐line treatments (PFS2/OS2, PFS3/OS3). A *p* < 0.05 was considered statistically significant. All analyses were performed in R version 3.5.1 (packages ‘smfcs’, ‘survminer’, ‘survival’, ‘gtsummary’ and ‘table1’ were used).^33^

## 3 RESULTS

### 3.1 Study population

**FIGURE 1:**
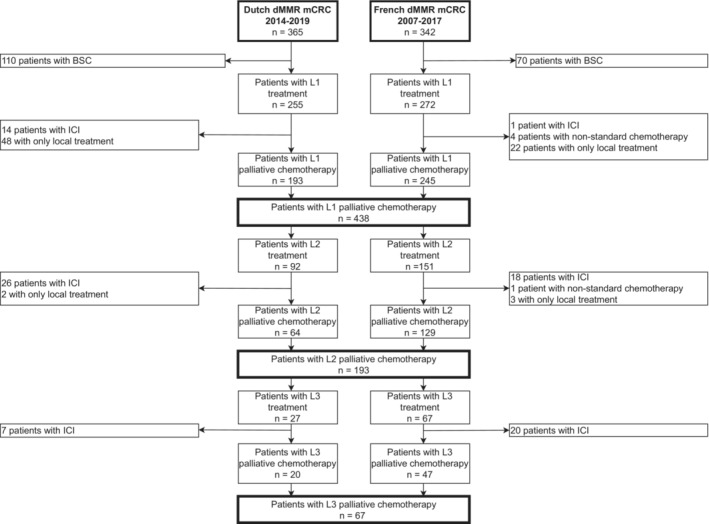
Flow diagram of Dutch and French dMMR/MSI‐H mCRC patients. BSC, best supportive care; dMMR, deficient mismatch repair system; ICIs, immune checkpoint inhibitors; L1, first‐line; L2, second‐line; L3, third‐line; mCRC, metastatic colorectal cancer.

The combined Dutch and French cohorts included a total of 707 patients of which 180 (25%) patients received best supportive care alone (BSC, Table S1) and 527 at least a first‐line treatment. A total of 438 patients received first‐line standard palliative chemotherapy with or without targeted therapy (1 L, 62%), 193 a standard second‐line treatment (2 L, 27%) and 67 a standard third‐line treatment (3 L, 9%) (Figure 1).

### 3.2 Best supportive care only

Patients receiving BSC only (*n* = 180) had a mean age of 74.6 years, 12% had proven or suspected Lynch syndrome, 47% had a WHO PS status of 2+ and tumors harbored a *BRAF* ^*V600E*^ mutation in 75% and a *RAS* mutation in 10% of cases (Table S1). Median OS was 2.9 months (95% CI: 2.5–3.7 months) for patients with BSC.

### 3.3 Population treated with first‐line palliative treatment

Patients receiving palliative first‐line chemotherapy with or without targeted therapy had a mean age of 61.9 years and 40% had proven or suspected Lynch syndrome. Tumors harbored a *BRAF* ^*V600E*^ mutation in 47%, a *RAS* mutation in 30% and a concomitant *BRAF* ^*V600E*^ and RAS mutation in 1% of cases (Table 1). The majority of sporadic dMMR/MSI‐H mCRC tumors harbored a *BRAF* ^*V600E*^ mutation (75%), while Lynch dMMR/MSI‐H tumors more often harbored a *RAS* mutation (65%) (Figure S1). Most patients with first‐line treatment received oxaliplatin‐based therapy (51%) or irinotecan‐based treatment (27%), which was combined with targeted therapy in 52% of patients, anti‐vascular endothelial growth factor (anti‐VEGF) (41%) or anti‐epidermal growth factor receptor (anti‐EGFR) (11%). In the French cohort more patients were treated with palliative first‐line chemotherapy with or without targeted therapy (72%) compared to the Dutch cohort (53%) and more patients were identified with proven or suspected Lynch syndrome (52% vs. 24%, *p* < 0.001) compared to the Dutch cohort (patient characteristics available in Table S2). Median follow‐up time for patients receiving palliative first‐line chemotherapy was 41.2 months for the total cohort (IQR 24.2–56.9 months), for the French cohort 38.1 months (IQR 20.4–66.8 months) and for the Dutch cohort 42.3 months (IQR 30.4–49.9 months). At the end of follow‐up 66% of patients were deceased.

**TABLE 1:** Patient, tumor, and treatment characteristics.

	First‐line palliative chemotherapy +/− targeted therapy (*N* = 438)
Age in years	
Mean (SD)	61.9 (14.6)
Sex	
Male	216 (49%)
Female	222 (51%)
Nationality	
Dutch	193 (44%)
French	245 (56%)
Sidedness	
Right‐sided	319 (74%)
Left‐sided	83 (19%)
Rectosigmoid/Rectum	31 (7%)
Missing	5
T‐stage	
T1–3	217 (54%)
T4	184 (46%)
Missing	37
N‐stage	
N0	104 (26%)
N1/2	300 (74%)
Missing	34
Resection status of primary tumor	
Resection	360 (82%)
No resection	78 (18%)
Differentiation grade	
Moderate/well	205 (56%)
Poor	161 (44%)
Missing	72
Adjuvant therapy	
Adjuvant therapy	136 (31%)
No adjuvant therapy	302 (69%)
Timing of metastases	
Synchronous	272 (62%)
Metachronous	166 (38%)
Number of metastatic sites	
1	271 (62%)
2 or more	167 (38%)
Liver involvement	
Liver involvement	196 (45%)
No liver involvement	242 (55%)
Peritoneal involvement	
Peritoneal involvement	176 (40%)
No peritoneal involvement	262 (60%)
*BRAF* ^*V600E*^ */RAS* status	
*BRAF* ^*V600E*^ mutation	160 (47%)
*RAS* mutation	101 (30%)
*BRAF* ^*V600E*^ and *RAS* wildtype	73 (22%)
*BRAF* ^*V600E*^ and *RAS* mutation	5 (1%)
Missing	94
Lynch syndrome status	
Lynch syndrome (proven or suspected)	148 (40%)
Sporadic case	223 (60%)
Missing	67
WHO performance status	
0–1	237 (84%)
2 or more	45 (16%)
Missing	156
Curative local treatment	
Curative local treatment	127 (29%)
No curative local treatment	311 (71%)
First‐line chemotherapy regimen	
Oxaliplatin‐based	222 (51%)
Irinotecan‐based	119 (27%)
Oxaliplatin and irinotecan‐based	21 (5%)
Cap/5‐FU alone	71 (16%)
Other	5 (1%)
First‐line targeted therapy	
Anti‐VEGF	181 (41%)
Anti‐EGFR	49 (11%)
No targeted therapy	208 (48%)

Abbreviations: CAP/5‐FU, capecitabine/5‐fluorouracil; EGFR, epidermal growth factor receptor; mCRC, metastatic colorectal cancer; N, nodal; SD, standard deviation; T, tumor; VEGF, vascular endothelial growth factor; WHO, World Health Organization.

**FIGURE 2:**
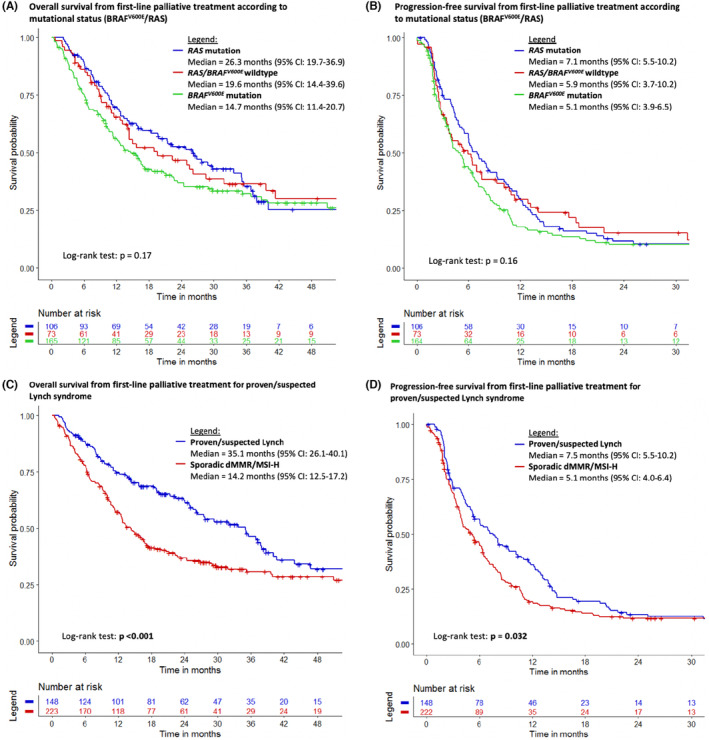
Kaplan–Meier curves of the overall and progression‐free survivals according to *RAS* mutation versus *BRAF* ^*V600E*^ mutation versus *RAS* and *BRAF* ^*V600E*^ wildtype (panels A and B), overall and progression‐free survivals according to Lynch syndrome versus sporadic dMMR/MSI‐H mCRC (panels C and D).

Median OS1 was 19.3 months (95% CI: 15.8–24.4) and median PFS1 6.0 months (95% CI: 5.0–6.7) for mCRC patients with dMMR/MSI‐H mCRC treated with palliative first‐line chemotherapy. Median OS1 was 14.7 months (95% CI: 11.4–20.7 months) for tumors harboring a *BRAF* ^*V600E*^ mutation, 26.3 months (95% CI: 19.7–36.9 months) for tumors harboring a *RAS* mutation, and 19.6 months (95% CI: 14.4–39.6 months) for *RAS* and *BRAF* ^*V600E*^ wildtype (Figure 2, *p* = 0.17). Median PFS1 was 5.1 months (95% CI: 3.9–6.5 months), 7.1 months (95% CI: 3.7–10.2 months) and 5.9 months (95% CI: 3.7–10.2 months), respectively (*p* = 0.16). Median OS1 and PFS1 for Lynch dMMR/MSI‐H mCRC patients were 35.1 months (95% CI: 26.1–40.1 months) and 7.5 months (95% CI: 5.5–10.2 months) versus 14.2 months (95% CI: 12.5–17.2 months, *p* < 0.001) and 5.1 months (95% CI: 4.0–6.4 months, *p* = 0.032) for sporadic dMMR/MSI‐H mCRC patients. Median OS1 for young (<60 years) and elder (≥60 years) dMMR/MSI‐H mCRC patients stratified for Lynch syndrome status did not show significant outcomes (*p* = 0.7 and *p* = 0.2, respectively) (Figure S2).

In patients with dMMR/MSI‐H mCRC treated with a palliative first‐line systemic treatment a total of 106 *RAS* mutations were observed, including 101 *KRAS* mutations and 5 *NRAS* mutations. *KRAS* mutations were most often G13D (27%), G12D (25%) or A146 (20%) (Figure S3). Median OS was 12.9 months (95% CI: 7.6 months‐NR) for patients harboring a *KRAS* A146 mutation compared to 25.1 months (95% CI: 16.7–40.1 months, *p* = 0.033) in patients with other *KRAS* mutations (Figure S4).

### 3.4 Prognostic factors

In multivariable regression analyses higher age, higher N‐stage, liver involvement and a WHO PS of two or higher were associated with worse OS in dMMR/MSI‐H mCRC patients treated with first‐line chemotherapy with or without targeted therapy (Table 2), while resection of primary tumor, a well‐differentiated tumor and irinotecan‐based doublet therapy were associated with better OS. Lynch syndrome was associated with survival in univariate analysis, but not in multivariate analysis. A higher number of metastatic sites (≥2 vs. 1) was significantly associated with worse PFS and female sex and primary tumor resection were associated with prolonged PFS (Table S3).

**TABLE 2:** Univariable and multivariable analyses of overall survival from start of first‐line palliative chemotherapy ± targeted therapy.

Characteristic	Category		Univariable regression				Multivariable regression		
			*N*	HR	95% CI	*p*‐value	HR	95% CI	*p*‐value
Age			438	1.03	1.02, 1.0	<0001	1.02	1.01, 1.04	<0.001
Sex	Female	(vs. male)	438	1.03	0.81, 1.31	0.8	0.89	0.67, 1.18	0.4
Sidedness	Left‐sided	(vs. right‐sided)	433	0.88	0.64, 1.20	0.4	1.05	0.74, 1.50	0.8
	Rectosigmoid/Rectum	(vs. right‐sided)	433	0.86	0.54, 1.35	0.5	1.19	0.70, 2.02	0.5
Resection status of primary tumor	Resection	(vs. no resection)	438	0.51	0.38, 0.68	<0.001	0.55	0.38, 0.80	0.002
Grade	Moderate/well	(vs. poor)	366	0.70	0.54, 0.92	0.009	0.74	0.55, 0.99	0.041
T‐stage	T4	(vs. T1–3)	401	1.27	0.99, 1.63	0.063	1.29	0.97, 1.71	0.075
N‐stage	N1–2	(vs. N0)	404	1.64	1.21, 2.24	0.002	1.72	1.20, 2.47	0.003
Adjuvant therapy	Received	(vs. not received)	438	0.94	0.72, 1.22	0.6	1.35	0.89, 2.03	0.2
Timing of metastases	Synchronous	(vs. metachronous)	438	1.16	0.90, 1.48	0.3	0.92	0.63, 1.36	0.7
Number of metastatic sites	2 or more	(vs. 1)	438	1.64	1.28, 2.09	<0.001	1.28	0.95, 1.73	0.10
Liver involvement	Yes	(vs. no involvement)	438	1.34	1.05, 1.70	0.017	1.40	1.04, 1.87	0.026
Peritoneal involvement	Yes	(vs. no involvement)	438	1.11	0.87, 1.41	0.4	1.17	0.88, 1.56	0.3
*BRAF* ^*V600E*^/RAS mutation status	*BRAF* ^*V600E*^ mutation	(vs. BRAF^V600E^/RAS wildtype)	391	1.25	0.88, 1.77	0.2	1.02	0.67, 1.54	>0.9
	*RAS* mutation	(vs. BRAF^V600E^/RAS wildtype)	391	0.91	0.61, 1.34	0.6	1.01	0.64, 1.59	>0.9
Lynch status	Sporadic dMMR/MSI‐H	(vs. Lynch)	371	1.61	1.22, 2.13	<0.001	1.07	0.66, 1.72	0.8
WHO performance score	2 or more	(vs. 0–1)	282	2.04	1.41, 2.94	<0.001	1.67	1.04, 2.67	0.035
Chemotherapy regimen	Doublet—oxaliplatin‐based	(vs. mono)	438	0.59	0.44, 0.80	<0.001	1.01	0.71, 1.45	>0.9
	Doublet—irinotecan‐based	(vs. mono)	438	0.46	0.32, 0.66	<0.001	0.62	0.39, 0.97	0.038
	Triplet	(vs. mono)	438	0.43	0.21, 0.87	0.019	0.84	0.39, 1.83	0.7
Targeted therapy	Anti‐EGFR	(vs. no targeted therapy)	438	1.02	0.68, 1.53	>0.9	1.20	0.74, 1.95	0.5
	Anti‐VEGF	(vs. no targeted therapy)	438	0.89	0.69, 1.15	0.4	0.97	0.73, 1.30	0.9

Abbreviations: CI, confidence interval; dMMR, deficient mismatch repair; EGFR, epidermal growth factor receptor; HR, hazard rate; mCRC, metastatic colorectal cancer; MSI‐H, microsatellite instability‐high; N, nodal; SD, standard deviation; T, tumor; VEGF, vascular endothelial growth; WHO, World Health Organization.

### 3.5 Line of treatment and chemosensitivity analyses

Median PFS1 for patients treated with a first‐line palliative chemotherapy was 6.0 months (95% CI: 5.0–6.7 months), for second‐line 3.8 months (95% CI: 3.1–4.4 months) and for third‐line 3.6 months (95% CI: 2.3–5.1 months) (Table 3). Chemotherapy +/− targeted therapy regimens for each line of treatment are available in the Table S4. Chemosensitivity analyses for PFS do not show significant results with regard to preference of chemotherapy or targeted therapy in the overall population or in different subgroups of the population (Table 4).

**TABLE 3:** Progression‐free survival and overall survival from diagnosis and consecutive lines of treatment.

From diagnosis	*N*	Median OS	Median PFS
	438	21.8 (95% CI 18.7–25.4)	–
Censored for immunotherapy in later lines	438	21.5 (95% CI 18.4–25.0)	–
Censored for curative surgery in later lines	438	18.8 (95% CI 16.7–22.1)	–
Censored for immunotherapy and/or curative surgery in later lines	438	18.5 (95% CI 16.7–21.8)	–
First‐line (OS1/PFS1)	438	19.3 (95% CI 15.8–24.4)	6.0 (95% CI 5.0–6.7)
Second‐line (OS2/PFS2)	193	11.7 (95% CI 10.7–15.9)	3.8 (95% CI 3.1–4.4)
Third‐line (OS3/PFS3)	67	8.8 (95% CI 6.8–13.4)	3.6 (95% CI 2.3–5.1)

Abbreviations: CI, confidence interval; OS, overall survival; OS1/OS2/OS3, overall survival from start of first‐line, second‐line or third‐line; PFS, progression‐free survival; PFS1/PFS2/PFS3, progression‐free survival from start of first‐line, second‐line or third line.

**TABLE 4:** Chemosensitivity analyses.

Chemosensitivity based on first‐line progression‐free survival (PFS1)						
	*N*	Unadjusted	*p*‐value	Multivariable cox adjusted model^a^		*p*‐value
		Median PFS1 in months (95% CI)		HR	95% CI	
Chemotherapy						
Whole population						
Oxaliplatin	222	5.5 (4.6–6.9)		1	–	
Irinotecan	119	6.5 (5.2–10.6)	0.3	0.79	0.57–1.11	0.5
BRAF^V600E^ mutation						
Oxaliplatin	83	5.3 (3.8–6.9)		1	–	
Irinotecan	39	4.8 (3.6–10.6)	0.8	0.94	0.58–1.53	0.8
RAS mutation						
Oxaliplatin	54	6.5 (5.3–10.3)		1	–	
Irinotecan	32	8.7 (6.1–14.3)	0.4	0.76	0.45–1.28	0.3
BRAF^V600E^/RAS wildtype						
Oxaliplatin	37	3.7 (2.8–9.3)		1	–	
Irinotecan	26	7.6 (5.2–18.8)	0.4	0.94	0.50–1.74	0.8
Lynch syndrome						
Oxaliplatin	69	5.5 (4.6–8.7)		1	–	
Irinotecan	55	10.2 (6.1–14.0)	0.2	0.83	0.56–1.24	0.4
Sporadic dMMR/MSI‐H						
Oxaliplatin	115	5.5 (3.9–6.9)		1	–	
Irinotecan	51	5.4 (3.6–1.6)	>0.9	1.03	0.70–1.54	0.9
Left‐sided						
Oxaliplatin	59	5.5 (4.6–8.3)		1	–	
Irinotecan	30	10.1 (6.5–14.2)	0.09	0.71	0.42–1.20	0.2
Right‐sided						
Oxaliplatin	158	5.4 (4.1–7.1)		1	–	
Irinotecan	89	5.4 (4.0–10.8)	0.8	0.92	0.67–1.27	0.6
Targeted therapy						
Whole population						
Anti‐EGFR	49	5.4 (2.9–12.2)		1	–	
Anti‐VEGF	181	6.5 (5.1–8.5)	0.7	1.03	0.65–1.63	>0.9
RAS wildtype^b^						
Anti‐EGFR	32	6.5 (3.9–14.0)		1	–	
Anti‐VEGF	83	5.1 (3.8–8.5)	0.3	1.02	0.58–1.79	>0.9
Lynch syndrome						
Anti‐EGFR	22	9.4 (2.9–21.7)		1	–	
Anti‐VEGF	66	9.3 (6.0–12.6)	0.8	0.86	0.47–1.58	0.6
Sporadic dMMR/MSI‐H						
Anti‐EGFR	21	5.4 (3.6–22.3)		1	–	
Anti‐VEGF	91	5.4 (4.1–7.6)	0.7	0.93	0.50–1.71	0.8
Right‐sided^b^						
Anti‐EGFR	40	6.1 (2.6–12.2)		1	–	
Anti‐VEGF	134	5.4 (4.2–8.5)	>0.9	0.88	0.56–1.38	0.6

Abbreviations: CI, confidence interval; dMMR, deficient mismatch repair; EGFR, epidermal growth factor receptor; MSI‐H, high microsatellite instability; PFS1, progression‐free survival from start of first‐line; VEGF, vascular endothelial growth factor.

^a^ Corrected for sex, primary tumor resection and number of metastatic sites. These are the significant variables in multivariable anlaysis on PFS1 in the total cohort (Table S3).

^b^ Sample size too small for *BRAF* ^*V600E*^ mutation, *RAS* mutation, *BRAF* ^*V600E*^ and *RAS* wildtype or left‐sided analyses.

## 4 DISCUSSION

To our knowledge, we present the largest real‐world data cohort of 707 dMMR/MSI‐H mCRC patients. Multivariable regression analysis of 438 treated patients with first‐line palliative chemotherapy showed that neither a *BRAF* ^*V600E*^ mutation, a *RAS* mutation or Lynch syndrome significantly affects OS or PFS in dMMR/MSI‐H mCRC patients. In addition, we did not show higher efficacy of a specific chemotherapy and/or targeted therapy regimens on PFS.

Differences between the Dutch and French cohorts were present such as differences in age (mean 65.4 years vs. 59.1 years), resection status of primary tumor (72% vs. 91%) and Lynch syndrome (24% vs. 52%), potentially due to differences in country guidelines or patient selection; in France patients were selected in expert centers and in the Netherlands patients were selected from all centers.

The median OS for patients with first‐line systemic therapy was 19.3 months from the start of first‐line treatment in our study. The median OS from other studies differed from 9 months to 39 months. A direct comparison to these studies is difficult due to differences in patient characteristics such as the number of patients with Lynch syndrome, inclusion of patients with initially resectable disease, administration of ICI and inclusion of trial or cohort patients.^4^, ^13^, ^15^, ^16^, ^25^, ^34^, ^35^, ^36^, ^37^

The median PFS for patients with a first‐line of palliative systemic therapy in our study was 6.0 months, which was comparable to other studies on dMMR/MSI‐H mCRC with a PFS varying from 4 to 6 months in dMMR/MSI‐H mCRC,^4^, ^15^, ^17^, ^34^, ^35^ but lower than PFS observed in pMMR/MSS mCRC ranging from 8 to 11 months.^38^, ^39^, ^40^, ^41^

Our study is in agreement with most studies showing no inferior prognosis for tumors with a *BRAF* ^*V600E*^ mutation in patients dMMR/MSI‐H mCRC.^15^, ^17^, ^18^, ^25^ By contrast three studies suggest *BRAF* ^*V600E*^ mutation as a driver for poor prognosis in dMMR/MSI‐H patients.^4^, ^11^, ^14^ However, in contrast to the latter studies, our study had a large sample size and obtained a multivariable analysis, including important variables as age, Lynch syndrome status and other relevant factors, which have a strong correlation with patients with *BRAF* ^*V600E*^ tumors and an important impact on the prognosis.^4^, ^13^, ^16^

Patients with tumors harboring a *RAS* mutation showed a trend towards better prognosis in univariate analysis, which is remarkable compared to the known inferior prognosis of *RAS* mutations in pMMR/MSS mCRC patients.^10^ However, in dMMR/MSI‐H mCRC patients this could also be explained by the strong association between patients with *RAS*‐mutated tumors and Lynch syndrome and younger age. The Lynch population is relatively young, which could be an important driver for better survival. The univariate effect is mitigated in multivariable analysis, with no significant effect on prognosis, consistent with other studies.^15^, ^18^, ^34^ The distribution of *KRAS* mutations in dMMR/MSI‐H mCRC is different compared to pMMR/MSS with relatively fewer *KRAS* codon 12 mutations and more *KRAS* A146 mutations.^42^ When studying specific *KRAS* mutations, *KRAS A146* mutation has been suggested as a distinct molecular subgroup with worse clinical outcomes and its underlying exon with mucinous/rare histological subtype.^43^, ^44^ In our study, we also show a shorter OS with *KRAS* A146 mutation, although the sample size is small (*N* = 11).

Lynch syndrome showed a superior survival in univariable analyses but not in multivariable analysis, potentially also due to the strong correlation between Lynch syndrome and young age. This is in agreement with other studies.^15^, ^18^ The strong association between *RAS* mutational status, age and Lynch syndrome could be of important knowledge when investigating subgroups in dMMR/MSI‐H mCRC patients. A subgroup of patients with a tumor harboring a *RAS* mutation did not show a significant survival increase in KEYNOTE‐177 to either pembrolizumab or chemotherapy.^45^ This subgroup of only 74 patients could include a high proportion of Lynch syndrome patients, who have a different natural history compared to sporadic dMMR/MSI‐H mCRC patients. A study of 466 patients with dMMR/MSI‐H mCRC treated with ICI did not show a significant association between *BRAF* ^*V600E*^ mutation, RAS mutation or Lynch syndrome on OS, however more research is warranted in this matter.^46^, ^47^

The effect of different treatment regimens on survival is uncertain in patients with dMMR/MSI‐H mCRC. No randomized controlled trial has been conducted to primarily analyze efficacy of different treatment regimens in dMMR/MSI‐H mCRC patients, and subgroups in cohort studies are often small due to the low incidence of dMMR/MSI‐H tumors in metastatic setting. Tougeron et al. have reported the largest series on chemosensitivity and presented no significant differences in chemotherapy and/or targeted therapy regimens, although a trend was seen in favor of anti‐VEGF (*n* = 67) compared to anti‐EGFR (*n* = 36).^15^ The main limitation of this study is the relatively small sample size which could induce impreciseness in subgroup analyses. These patients are included in our current cohort (56% of first‐line patients are from the French cohort), resulting in overlapping results, but the large number of patients in the current study allowed more robust analyses concerning efficacy of the chemotherapy and targeted therapy regimens. We do not show a higher efficacy of a specific chemotherapy (irinotecan vs. oxaliplatin‐based chemotherapy) or targeted therapy (anti‐VEGF vs. anti‐EGFR) regimen.

It is worthy to note that one fourth of the patients were treated with BSC alone. As expected, these patients were older, with poor performance status and were therefore likely to be unfit for conventional chemotherapy, although it could also be the choice of the patient. The inclusion period of our cohort was mainly in the pre‐immunotherapy era and a subset of these patients might nowadays be eligible for immunotherapy. Consequently, it is of importance to generate data about the efficacy and tolerability of ICI for elderly/frail patients with dMMR/MSI‐H mCRC.^48^, ^49^

Strengths of this study are the largest sample size of dMMR/MSI‐H mCRC patients in the pre‐immunotherapy era up until now and the high‐quality data, including knowledge of *BRAF* ^*V600E*^ and *RAS* mutations, proven/suspected Lynch syndrome status and consecutive regimens of chemotherapy with or without targeted therapy. One of the limitations is the retrospective nature of this study. Despite the retrospective nature we only had a small number of missing data and multiple imputation was used to address this issue. Regarding the use of different treatment regimens on PFS and OS, multivariable analyses included many relevant variables. However, due to the retrospective nature of the study there could be unknown confounding. MMR/MSI status was only known when determined in clinical practice, which could induce patient's selection bias. This could overestimate PFS and OS, since MMR/MSI status might not be determined in patients with a very poor prognosis. However, this limitation is potentially inconsequential since the cohort included 25% of patients with BSC alone. Finally, there were differences in patient, tumor and treatment characteristics between the Dutch and French cohort, however, these were accounted for by stratification for nationality in multivariable analyses.

## 5 CONCLUSION

In this largest high‐quality real‐world cohort to date, we observed that known factors as age and WHO performance score were significantly associated with OS in multivariable analysis, however that *BRAF* ^*V600E*^ and *RAS* mutational status are not associated with prognosis in dMMR/MSI‐H mCRC patients treated with palliative first‐line chemotherapy. This is in contrast to pMMR/MSS mCRC patients. Lynch syndrome is also not an independent prognostic factor for survival. No superior efficacious chemotherapy regimen and targeted agent could be identified. Our results show that the impact of molecular markers on prognostication can differ between subgroups of mCRC and these findings underline the complex heterogeneity of mCRC.

## AUTHOR CONTRIBUTIONS

**Koen Zwart:** Conceptualization (equal); data curation (equal); formal analysis (equal); investigation (equal); methodology (equal); project administration (equal); resources (equal); software (equal); validation (equal); visualization (equal); writing – original draft (equal); writing – review and editing (equal). **Frederieke H van der Baan:** Conceptualization (equal); data curation (equal); formal analysis (equal); investigation (equal); methodology (equal); project administration (equal); resources (equal); supervision (equal); validation (equal); writing – original draft (equal); writing – review and editing (equal). **Romain Cohen:** Writing – review and editing (equal). **Thomas Aparicio:** Writing – review and editing (equal). **Christelle De La Fouchardiere:** Writing – review and editing (equal). **Thierry Lecomte:** Writing – review and editing (equal). **Cornelis J.A. Punt:** Writing – review and editing (equal). **David Sefrioui:** Writing – review and editing (equal). **Rik Verheijden:** Methodology (equal); writing – review and editing (equal). **Geraldine Vink:** Conceptualization (equal); project administration (equal); resources (equal); supervision (equal); writing – review and editing (equal). **Emerens Wensink:** Writing – review and editing (equal). **Aziz Zaanan:** Writing – review and editing (equal). **M. Koopman:** Conceptualization (equal); supervision (equal); writing – review and editing (equal). **David Tougeron:** Conceptualization (equal); methodology (equal); project administration (equal); resources (equal); supervision (equal); writing – original draft (equal); writing – review and editing (equal). **Jeanine Roodhart:** Conceptualization (equal); funding acquisition (equal); methodology (equal); project administration (equal); supervision (equal); visualization (equal); writing – original draft (equal); writing – review and editing (equal).

## FUNDING INFORMATION

The author(s) received no specific funding for this work.

## CONFLICT OF INTEREST STATEMENT

AZ: Pierre Fabre, Merck, Baxter, Havas Life, Amgen, Roche. CdlF: None. CJP: Nordic Pharma. RC: Servier Institute, Nuovo‐Soldati Foundation, ARC Foundation for Cancer Research, MSD. DS: Servier, Ipsen, Astella, Roche. DT: Amgen, MSD, Pierre Fabre, Merck, Roche, Bayer, Ipsen. EW: None. FvdB: None. GV: BMS, Merck, Servier, Personal Genome Diagnostics, Bayer, Sirtex, Pierre Fabre, Lilly. JR: Bayer, BMS, Merck‐Serono, Pierre Fabre, Servier, HUB4organoids, Cleara Biotech. KZ: None. MK: Nordic Farma, Merck‐Serono, Pierre Fabre, Servier, Bayer, Bristol Myers Squibb, Merck, Roche and Servier. RV: None TL: Amgen, Servier, Sanofi. TA: Roche, Sirtec, Sanofi, Amgen.

## ETHICS APPROVAL AND CONSENT TO PARTICIPATE

Dutch data were pseudonomized and consent was obtained by an opt‐out approach. The French data collection was approved by the ethical committee *Comité de Protection des Personnes Ouest III* and, Due to the retrospective nature of the study and since most patients were deceased, informed consent was waived. The study was performed in accordance with the Declaration of Helsinki.

## Supporting information


Appendix S1.

## Data Availability

The data generated in this study are available upon request from the corresponding author.
