# Supplementary material for: Prognostic value of Lynch syndrome, BRAFV600E , and RAS mutational status in dMMR/MSI‐H metastatic colorectal cancer in a pooled analysis of Dutch and French cohorts
Source: Cancer Med. 2023 Jun 16;12(15):15841–53. doi: 10.1002/cam4.6223 (PMC10469760; doi:10.1002/cam4.6223)

**Supplemtary files**

**Definitions**

[Definition Lynch syndrome 2](#_Toc137477292)

**Tables**

[Table S1. Patient and tumor characteristics of patients with best supportive care only and with systemic and/or local treatment and best supportive care. 3](#_Toc137477293)

[Table S2. Patient and tumor characteristics of patients with first-line palliative systemic treatment stratified by country 5](#_Toc137477294)

[Table S3. Univariable and multivariable analyses of progression-free survival from start of first line chemotherapy +/- targeted therapy 7](#_Toc137477295)

[Table S4. Chemotherapy and targeted therapy regimens in first-line, second-line, and third-line patients. 9](#_Toc137477296)

**Figures**

[Figure S1. Molecular subgroups in sporadic dMMR/MSI-H and Lynch patients (numbers are rounded to nearest 5%). 10](#_Toc137477297)

[Figure S2. Overall survival for first-line patient <60 and ≥60 years stratified for Lynch syndrome status. 11](#_Toc137477298)

[Figure S4. Overall survival for first-line patients with KRAS mutation A146 versus non-A146 KRAS mutation. 13](#_Toc137477299)

# **Definition Lynch syndrome**

Lynch:

1. MMR germline mutation

Probable Lynch:

1. Loss of protein expression MSH6 or MSH2 or
2. Loss of protein expression of PMS2 without loss of MLH1 or
3. Loss of protein expression of MLH1 without a *BRAF^V600E^* mutation and without MLH1 promotor hypermethylation or
4. Loss of MLH1 expression without *BRAF^V600E^* mutation and unknown MLH1 promotor hypermethylation, however did meet the Amsterdam II criteria.

Probable sporadic:

1. Loss of MLH1 protein expression and *BRAF^V600E^* mutation or
2. Loss of MLH1 protein expression and MLH1 promotor hypermethylation.

# **Table S1.** Patient and tumor characteristics of patients with best supportive care only and with systemic and/or local treatment and best supportive care.

|  | **Best supportive care only (N=180)** | **Treatment and best supportive care (N=527)** |
| --- | --- | --- |
| **Age in years** |  |  |
| Mean (SD) | 74.6 (12.4) | 62.3 (14.6) |
| **Sex** |  |  |
| Male | 66 (37%) | 251 (48%) |
| Female | 114 (63%) | 276 (52%) |
| **Nationality** |  |  |
| Dutch | 110 (61%) | 255 (48%) |
| French | 70 (39%) | 272 (52%) |
| **Sidedness** |  |  |
| Right-sided | 146 (82%) | 390 (75%) |
| Left-sided | 28 (16%) | 96 (18%) |
| Rectosigmoid/Rectum | 5 (3%) | 33 (6%) |
| Missing | 1 | 8 |
| **Resection primary tumor** |  |  |
| Resection | 148 (82%) | 447 (85%) |
| No resection | 32 (18%) | 80 (15%) |
| **Differentiation grade** |  |  |
| Moderate/well | 68 (45%) | 248 (56%) |
| Poor | 84 (55%) | 195 (44%) |
| Missing | 28 | 84 |
| **Adjuvant therapy** |  |  |
| Adjuvant therapy | 31 (17%) | 170 (32%) |
| No adjuvant therapy | 149 (83%) | 357 (68%) |
| **Timing of metastases** |  |  |
| Synchronous | 117 (65%) | 324 (61%) |
| Metachronous | 63 (35%) | 203 (39%) |
| **Number of metastatic sites** |  |  |
| 1 | 105 (59%) | 335 (64%) |
| 2 or more | 73 (41%) | 192 (36%) |
| Missing | 2 | 0 |
| **Liver involvement** |  |  |
| Liver involvement | 77 (43%) | 231 (44%) |
| No liver involvement | 103 (57%) | 296 (56%) |
| **Peritoneal involvement** |  |  |
| Peritoneal involvement | 77 (43%) | 217 (41%) |
| No peritoneal involvement | 103 (57%) | 310 (59%) |
| ***BRAF^V600E^/RAS* status** |  |  |
| *BRAF^V600E^* mutation | 69 (75%) | 186 (48%) |
| *RAS* mutation | 9 (10%) | 109 (28%) |
| *BRAF^V600E^* and *RAS* wildtype | 11 (12%) | 83 (22%) |
| *BRAF^V600E^* and *RAS* mutation | 3 (3%) | 6 (2%) |
| Missing | 88 | 143 |
| **Lynch syndrome status** |  |  |
| Lynch syndrome (proven or suspected) | 16 (12%) | 172 (39%) |
| Sporadic case | 112 (88%) | 273 (61%) |
| Missing | 52 | 82 |
| **WHO performance status** |  |  |
| 0-1 | 55 (53%) | 278 (84%) |
| 2 or more | 49 (47%) | 53 (16%) |
| Missing | 76 | 196 |
|  |  |  |
| Abbreviations: SD = standard deviation; WHO = World health organization | | |

# **Table S2.** Patient and tumor characteristics of patients with first-line palliative systemic treatment stratified by country

|  | **Dutch (N=193)** | **French (N=245)** | **P-value** |
| --- | --- | --- | --- |
| **Age in years** |  |  |  |
| Mean (SD) | 65.4 (12.1) | 59.1 (15.8) | **<0.001** |
| **Sex** |  |  |  |
| Male | 89 (46%) | 127 (52%) | 0.3 |
| Female | 104 (54%) | 118 (48%) |  |
| **Sidedness** |  |  |  |
| Right-sided | 142 (76%) | 177 (72%) | 0.7 |
| Left-sided | 34 (18%) | 49 (20%) |  |
| Rectosigmoid/Rectum | 12 (6%) | 19 (8%) |  |
| Missing | 5 | 0 |  |
| **T-stage** |  |  |  |
| T1-3 | 98 (55%) | 119 (53%) | 0.9 |
| T4 | 80 (45%) | 104 (47%) |  |
| Missing | 15 | 22 |  |
| **N-stage** |  |  |  |
| N0 | 46 (25%) | 58 (26%) | 0.4 |
| N1/2 | 136 (75%) | 164 (74%) |  |
| Missing | 11 | 23 |  |
| **Resection status of primary tumor** |  |  |  |
| Resection | 138 (72%) | 222 (91%) | <**0.001** |
| No resection | 55 (28%) | 23 (9%) |  |
| **Differentiation grade** |  |  |  |
| Moderate/well | 74 (50%) | 131 (60%) | 0.070 |
| Poor | 74 (50%) | 87 (40%) |  |
| Missing | 45 | 27 |  |
| **Adjuvant therapy** |  |  |  |
| Adjuvant therapy | 39 (20%) | 97 (40%) | **<0.001** |
| No adjuvant therapy | 154 (80%) | 148 (60%) |  |
| **Timing of metastases** |  |  |  |
| Synchronous | 136 (70%) | 136 (56%) | **0.002** |
| Metachronous | 57 (30%) | 109 (44%) |  |
| **Number of metastatic sites** |  |  |  |
| 1 | 108 (56%) | 163 (67%) | **0.031** |
| 2 or more | 85 (44%) | 82 (33%) |  |
| **Liver involvement** |  |  |  |
| Liver involvement | 89 (46%) | 107 (44%) | 0.7 |
| No liver involvement | 104 (54%) | 138 (56%) |  |
| **Peritoneal involvement** |  |  |  |
| Peritoneal involvement | 68 (35%) | 108 (44%) | 0.076 |
| No peritoneal involvement | 125 (65%) | 137 (56%) |  |
| ***BRAF^V600E^/RAS* status** |  |  |  |
| *BRAF^V600E^* mutation | 81 (53%) | 79 (42%) | **0.005** |
| *RAS* mutation | 32 (21%) | 69 (37%) |  |
| *BRAF^V600E^* and *RAS* wildtype | 37 (24%) | 36 (19%) |  |
| *BRAF^V600E^* and *RAS* mutation | 3 (2%) | 2 (1%) |  |
| Missing | 40 | 59 |  |
| **Lynch syndrome status** |  |  |  |
| Lynch syndrome (proven or suspected) | 38 (24%) | 110 (52%) | **<0.001** |
| Sporadic case | 121 (76%) | 102 (48%) |  |
| Missing | 34 | 33 |  |
| **WHO performance status** |  |  |  |
| 0-1 | 104 (86%) | 133 (83%) | 0.6 |
| 2 or more | 17 (14%) | 28 (17%) |  |
| Missing | 72 | 84 |  |
| **Curative local treatment** |  |  |  |
| Curative local treatment | 53 (27%) | 74 (30%) | 0.6 |
| No curative local treatment | 140 (73%) | 171 (70%) |  |
| **First-line chemotherapy regimen** |  |  |  |
| Oxaliplatin | 110 (57%) | 112 (46%) | **<0.001** |
| Irinotecan | 26 (13%) | 93 (38%) |  |
| Oxaliplatin and irinotecan | 8 (4%) | 13 (5%) |  |
| Cap/5-FU | 46 (24%) | 25 (10%) |  |
| Other | 3 (2%) | 2 (1%) |  |
| **First-line targeted therapy** |  |  |  |
| Anti-VEGF | 101 (52%) | 80 (33%) | **<0.001** |
| Anti-EGFR | 4 (2%) | 45 (18%) |  |
| No targeted therapy | 88 (46%) | 120 (49%) |  |
|  |  |  |  |
| Abbreviations: CAP/5-FU = capecitabine/5-fluorouracil; EGFR = epidermal growth factor receptor; mCRC = metastatic colorectal cancer; SD = standard deviation; VEGF = vascular endothelial growth factor; WHO = world health organization | | | |

# **Table S3.** Univariable and multivariable analyses of progression-free survival from start of first line chemotherapy +/- targeted therapy

| **Characteristic** |  |  | **Univariable regression** | | | | **Multivariable regression** | | | |
| --- | --- | --- | --- | --- | --- | --- | --- | --- | --- | --- |
|  | **Categories** | **Reference group** | Total N | HR | 95% CI | p-value | | HR*^1^* | 95% CI | p-value |
| **Age** |  |  | 438 | 1.01 | 1.00, 1.02 | **0.012** | | 1.01 | 0.99, 1.02 | 0.3 |
| **Sex** | Female | *(versus male)* | 438 | 0.90 | 0.73, 1.11 | 0.3 | | 0.78 | 0.62, 0.99 | **0.044** |
| **Sidedness** | Left-sided | *(versus right-sided)* | 433 | 0.90 | 0.69, 1.18 | 0.5 | | 1.00 | 0.74, 1.34 | >0.9 |
|  | Rectosigmoid/Rectum | *(versus right-sided)* | 433 | 1.00 | 0.68, 1.48 | >0.9 | | 1.17 | 0.75, 1.81 | 0.5 |
| **Resection status of primary** | Resection | *(versus no resection)* | 438 | 0.58 | 0.44, 0.75 | **<0.001** | | 0.59 | 0.43, 0.82 | **0.002** |
| **Differentiation grade** | Moderate/well | *(versus poor)* | 366 | 0.94 | 0.74, 1.18 | 0.6 | | 0.91 | 0.70, 1.16 | 0.4 |
| **T-stage** | T4 | *(versus T1-3)* | 401 | 1.06 | 0.85, 1.32 | 0.6 | | 1.01 | 0.79, 1.30 | >0.9 |
| **N-stage** | N1-2 | *(versus N0)* | 404 | 1.21 | 0.94, 1.56 | 0.13 | | 1.19 | 0.89, 1.60 | 0.2 |
| **Adjuvant therapy** | Received | *(versus not received)* | 438 | 0.95 | 0.75, 1.19 | 0.6 | | 1.12 | 0.79, 1.59 | 0.5 |
| **Timing of metastases** | Synchronous | *(versus metachronous)* | 438 | 1.07 | 0.86, 1.33 | 0.5 | | 0.92 | 0.66, 1.27 | 0.6 |
| **Number of metastatic sites** | 2+ | *(versus 1)* | 438 | 1.83 | 1.48, 2.27 | **<0.001** | | 1.63 | 1.26, 2.11 | **<0.001** |
| **Liver involvement** | Yes | *(versus no involvement)* | 438 | 1.27 | 1.03, 1.56 | **0.027** | | 1.16 | 0.90, 1.50 | 0.2 |
| **Peritoneal involvement** | Yes | *(versus no involvement)* | 438 | 1.20 | 0.97, 1.48 | 0.092 | | 1.17 | 0.91, 1.51 | 0.2 |
| **BRAF^V600E^/RAS status** | *BRAF^V600E^* mutation | *(versus BRAF^V600E^ and RAS wildtype)* | 339 | 1.22 | 0.98, 1.52 | 0.075 | | 1.18 | 0.81, 1.70 | 0.4 |
|  | *RAS* mutation | *(versus BRAF^V600E^ and RAS wildtype)* | 339 | 0.93 | 0.67, 1.31 | 0.7 | | 1.01 | 0.69, 1.48 | >0.9 |
| **Lynch status** | Sporadic dMMR/MSI-H | *(versus Lynch)* | 371 | 1.28 | 1.02, 1.61 | **0.035** | | 1.17 | 0.77, 1.79 | 0.5 |
| **WHO performance score** | 2+ | *(versus 0-1)* | 282 | 1.56 | 1.11, 2.20 | **0.011** | | 1.32 | 0.90, 1.92 | 0.2 |
| **Chemotherapy regimen** | Doublet – oxaliplatin-based | *(versus mono)* | 438 | 0.90 | 0.68, 1.20 | 0.5 | | 1.15 | 0.83, 1.58 | 0.4 |
|  | Doublet – irinotecan-based | *(versus mono)* |  | 0.78 | 0.57, 1.08 | 0.13 | | 1.02 | 0.67, 1.55 | >0.9 |
|  | Triple | *(versus mono)* |  | 0.64 | 0.36, 1.13 | 0.13 | | 0.98 | 0.52, 1.86 | >0.9 |
| **Targeted therapy** | Anti-EGFR | *(versus no targeted therapy)* | 438 | 0.93 | 0.66, 1.31 | 0.7 | | 0.86 | 0.58, 1.30 | 0.5 |
|  | Anti-VEGF | *(versus no targeted therapy)* |  | 0.88 | 0.70, 1.09 | 0.2 | | 0.85 | 0.65, 1.11 | 0.2 |
| Abbreviations: CI = confidence interval; dMMR = deficient mismatch repair; EGFR = epidermal growth factor receptor; HR = hazard rate; mCRC = metastatic colorectal cancer; MSI-H = microsatellite instability-high; SD = standard deviation; VEGF = vascular endothelial growth factor; WHO = world health organization | | | | | | | | | | |

# **Table S4.** Chemotherapy and targeted therapy regimens in first-line, second-line, and third-line patients.

| **Line of therapy** | **N** |  |
| --- | --- | --- |
| **First-line (N=438)**  *Oxaliplatin-based*  *Irinotecan-based*  *Oxaliplatin + irinotecan*  *Only fluoropyrimidines*  *Other*  *Anti-VEGF*  *Anti-EGFR*  *No targeted therapy* | *222*  *119*  *21*  *71*  *5*  *181*  *49*  *208* | *(51%)*  *(27%)*  *(5%)*  *(16%)*  *(5%)*  *(41%)*  *(11%)*  *(48%)* |
| **Second-line (N=193)**  *Oxaliplatin-based*  *Irinotecan-based*  *Oxaliplatin + irinotecan*  *Only fluoropyrimidines*  *Other*  *Anti-VEGF*  *Anti-EGFR*  *No targeted therapy* | *46*  *109*  *5*  *13*  *20*  *83*  *37*  *73* | *(24%)*  *(57%)*  *(3%)*  *(7%)*  *(10%)*  *(43%)*  *(19%)*  *(38%)* |
| **Third-line (N=67)**  *Oxaliplatin-based*  *Irinotecan-based*  *Oxaliplatin + irinotecan*  *Only fluoropyrimidines*  *Other*  *Anti-VEGF*  *Anti-EGFR*  *No targeted therapy* | *8*  *34*  *2*  *2*  *21*  *13*  *27*  27 | *(12%)*  *(51%)*  *(3%)*  *(3%)*  *(31%)*  *(19%)*  *(40%)*  *(40%)* |

Abbreviations: EGFR = epidermal growth factor receptor; VEGF = vascular endothelial growth factor

# **Figure S1.** Molecular subgroups in sporadic dMMR/MSI-H and Lynch patients (numbers are rounded to nearest 5%).


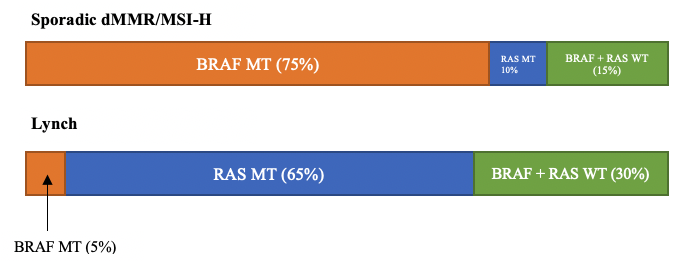


# **Figure S2.** Overall survival for first-line patient <60 and ≥60 years stratified for Lynch syndrome status.


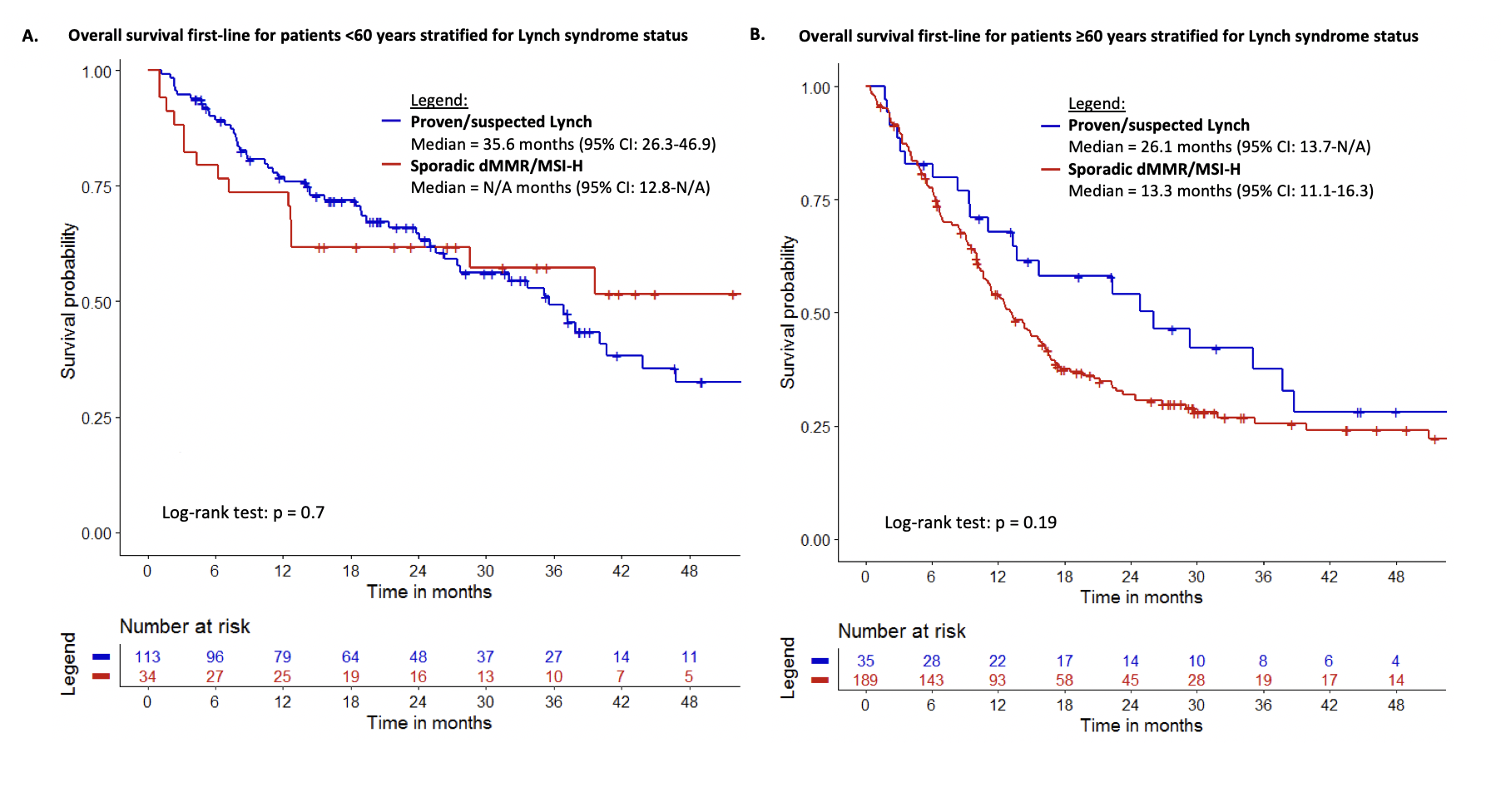
Figure S3. Specific RAS mutations in the dMMR/MSI-H patients with first-line systemic treatment


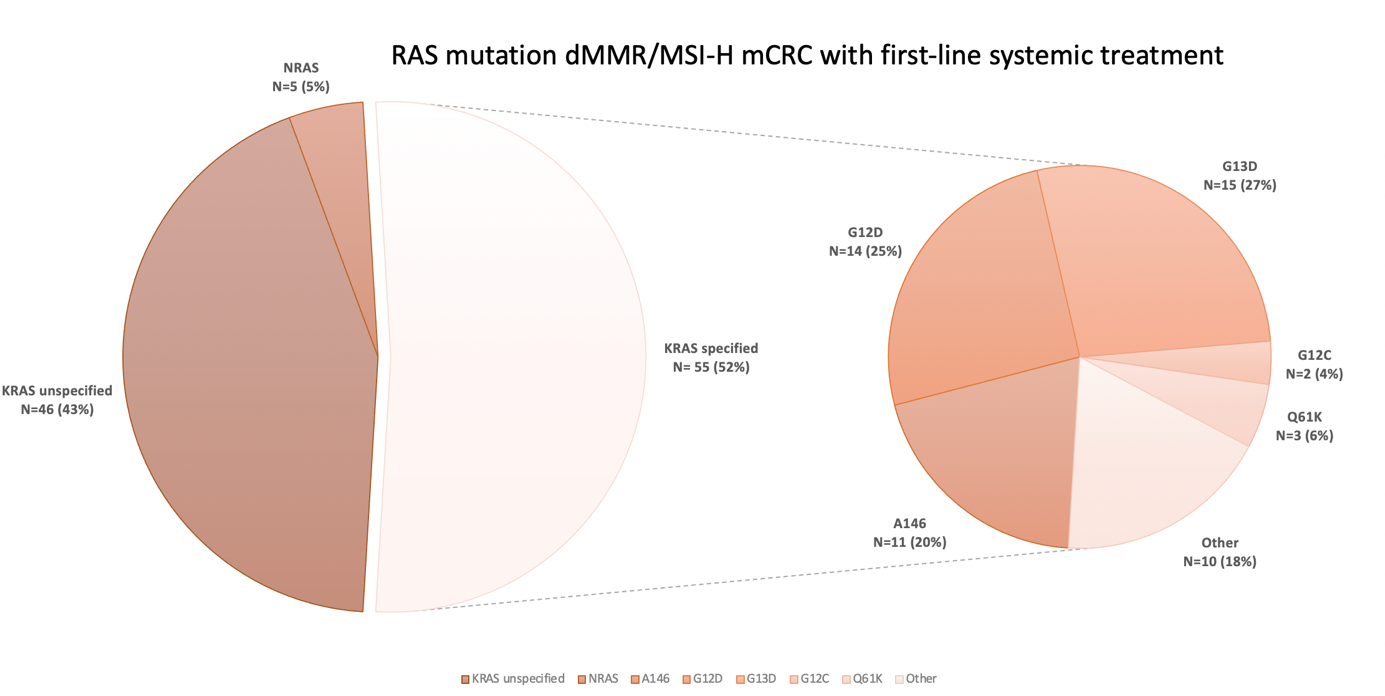


# **Figure S4.** Overall survival for first-line patients with KRAS mutation A146 versus non-A146 KRAS mutation.


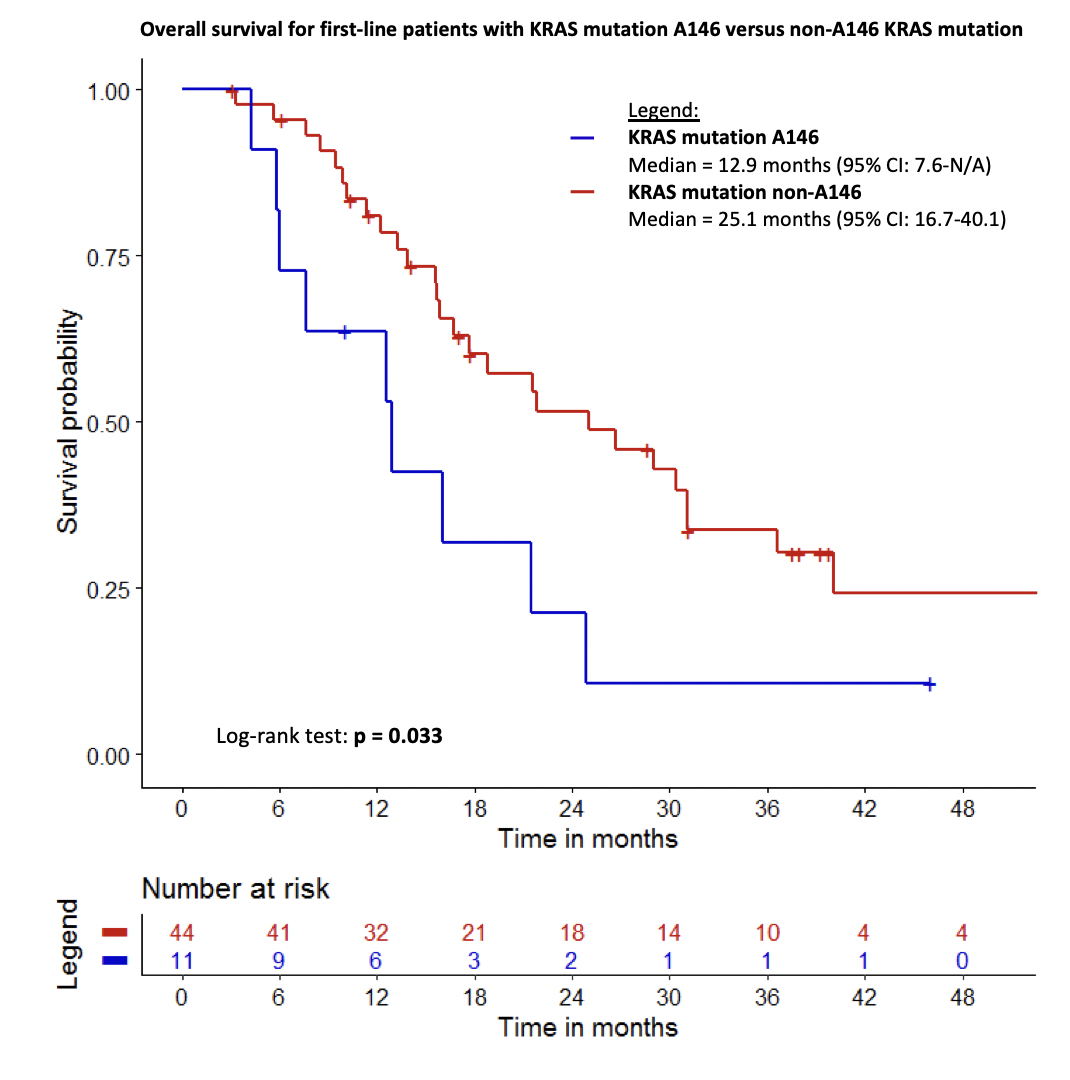

Supplement: Supplementary file 1 — Appendix S1. [file CAM4-12-15841-s001.docx]
